# Bilateral renal artery stenosis as a cause of refractory intradialytic hypertension in a patient with end stage renal disease

**DOI:** 10.1186/s12882-018-1191-z

**Published:** 2019-01-14

**Authors:** Zachary Wolfmueller, Kunal Goyal, Bhanu Prasad

**Affiliations:** 1 College of Medicine, University of Saskatchewan, Regina Campus, 1440, 14th Avenue, Regina, S4P 0W5 Canada; 2 Department of Radiology, Regina General Hospital, 1440, 14th Avenue, Regina, SK S4P 0W5 Canada; 3 Section of Nephrology, Department of Medicine, Regina General Hospital, 1440, 14th Avenue, Regina, S4P 0W5 Canada

## Abstract

**Background:**

We report a 61-year-old female with end-stage renal disease (ESRD) secondary to polycystic kidney disease (PKD) complicated by intradialytic hypertension (IDH). Increased sympathetic drive leading to increased stroke volume and/or vasoconstriction with an inappropriate increase in peripheral vascular resistance (PVR) has been postulated to be the cause of IDH.

**Case presentation:**

Attempts to control her blood pressure by reducing her goal weight; increasing dialysis times/ frequency and decreasing her sodium concentrate in the dialysis fluid were unsuccessful. Acting upon literature evidence suggesting renovascular disease as a cause of IDH, we referred her to an interventional radiologist for evaluation of the renal arteries. Selective angiogram of both renal arteries revealed right sided atherosclerotic renal artery stenosis (RAS) treated with insertion of a balloon mounted 6 mm stent and left sided fibromuscular dysplasia (FMD) treated with 5 mm balloon angioplasty.

**Conclusions:**

This case highlights the need for interrogating the renal arteries radiologically for a potential cause in difficult to control IDH and comments on the association between PKD and FMD that has not yet been reported.

## Background

Hypertension on renal replacement therapy (RRT) is best managed by optimizing goal weight, tight fluid control, salt restriction, increased dialysis times/frequency, decreasing sodium in the dialysate and transitioning to home hemodialysis. While ultrafiltration on hemodialysis typically lowers blood pressure, some patients have a paradoxical rise in blood pressure called intradialytic hypertension (IDH).

## Case presentation

A 61-year-old female was being actively monitored in the hemodialysis unit for intradialytic hypertension (IDH). She was born in Ethiopia and had been diagnosed with autosomal dominant polycystic kidney disease (ADPKD) at the age of 35 and immigrated to Canada at the age of 53. Her mother died in Ethiopia and with no access to medical facilities. Our patient was certain that her mother died of complications of hypertension, but couldn’t remember any specifics. Her brother had successfully received a kidney transplant for ADPKD. Her medical history was also significant for vertically transmitted hepatitis B, rheumatoid arthritis (high titre rheumatoid factor, 515 IU/ml and high titre anti-cyclic citrullinated peptide, 34 U/ml) and latent tuberculosis infection treated with 6 months of isoniazid and rifampin.

Ten years prior, she was initiated on a single agent for blood pressure (angiotensin receptor blocker) and 3 years after arrival to Canada progressed to ESRD. She was initiated on hemodialysis (HD) with a left arterio-venous (AV) fistula requiring three antihypertensives. Over the next 4 years, her blood pressure continued to worsen and more so intradialytically and required six agents for control. She weighed 55 kgs and was 172 cm tall with a body mass index (BMI) of 18.4. Her interdialytic weight gain was four kgs at a frequency of 3/week. She was not taking anti-inflammatories for pain relief as her rheumatoid arthritis was quiescent and was on 75 units/kg body weight of erythropoietin, which maintained the hemoglobin between 100 and 110 g/L. Her dialysate (mmol/L) consisted of sodium 135, potassium 2, bicarbonate 35 and calcium 1.25. The average interdialytic 24-h ambulatory blood pressure was 158/78 mmHg. We attempted to treat her with increasing the frequency of dialysis to 4/week, decreasing the dialysate sodium to 130 mmol, increasing the duration to 4.5 h, (led to inter dialytic weight gains of 2.5 kgs instead of 4 kgs) but made no impact on her blood pressure. Our attempts to decrease her goal weight further were limited by severe cramps. Aldosterone blockade with spironolactone had no noticeable improvement and she was unable to tolerate the sympatholytic agent clonidine. Due to insufficient control of her blood pressure on six medications the patient was referred to interventional radiology for evaluation of renovascular causes of hypertension. Prior to intervention, she was on hydralazine (100 mg qid), amlodipine (10 mg od), telmisartan (80 mg od), labetalol (200 mg tid), furosemide (80 mg od) and amiloride (5 mg od).

**Fig. 1:**
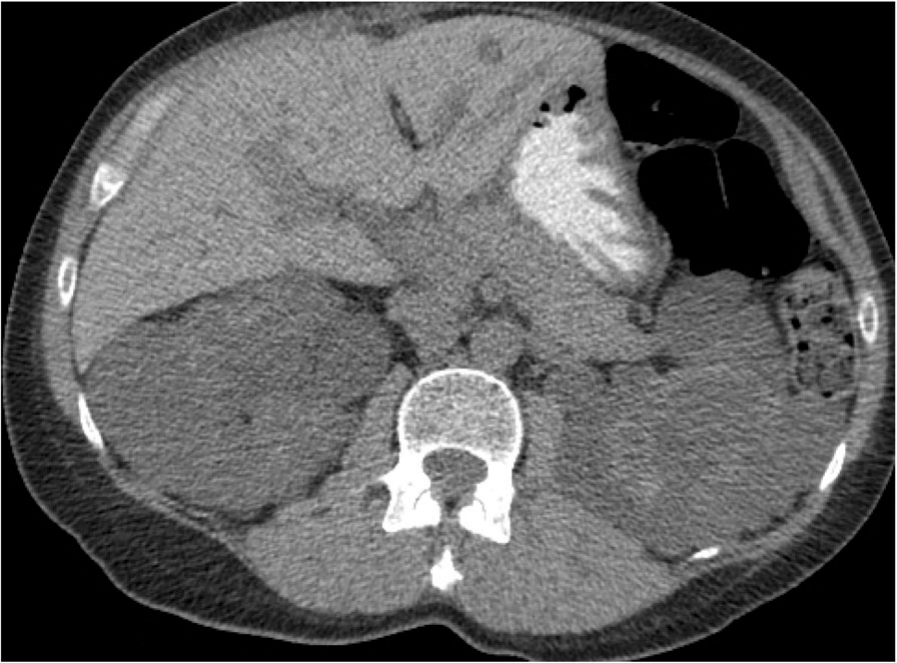
Unenhanced axial image shows typical appearances of PCKD involving both kidneys

**Fig. 2:**
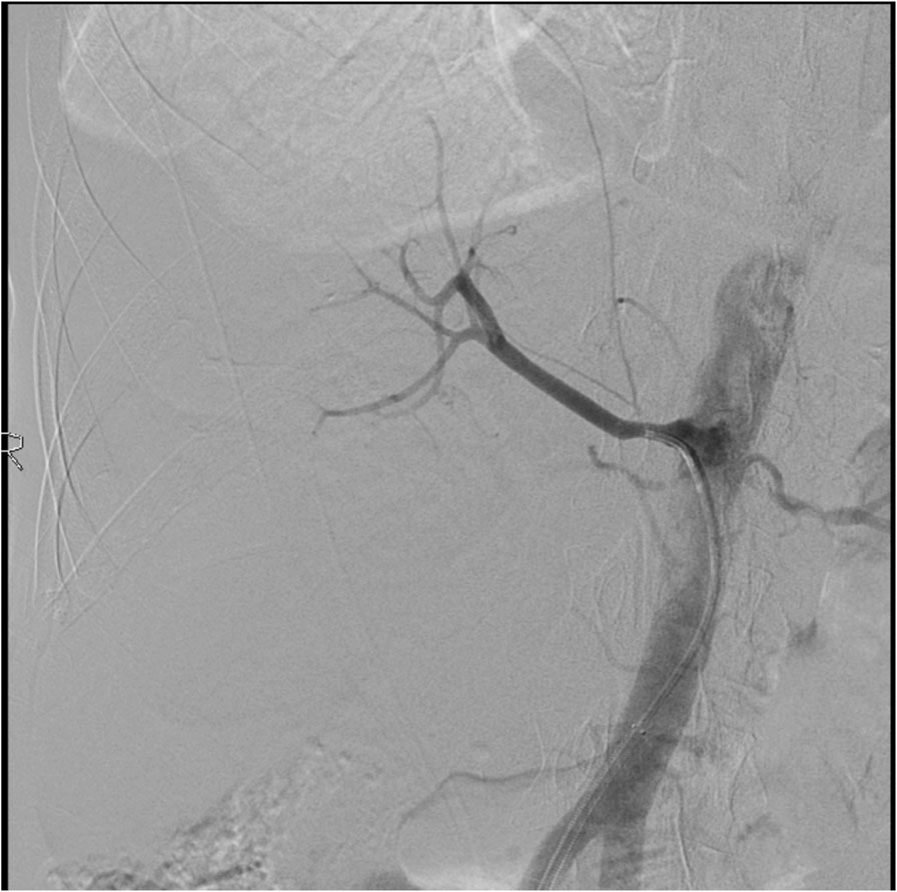
Selective right renal angiogram showing focal moderate severity stenosis at the ostium

**Fig. 3:**
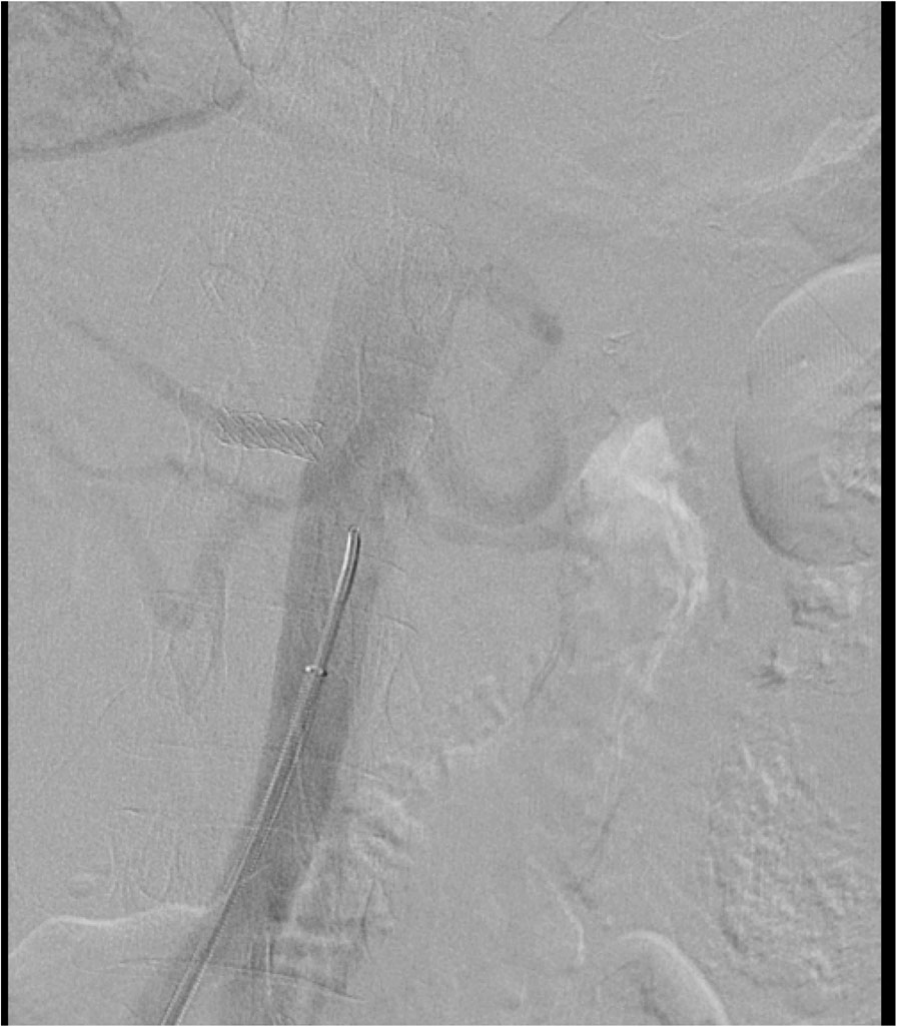
Post stent insertion angiogram showing satisfactory resolution of the right renal artery ostial stenosis

**Fig. 4:**
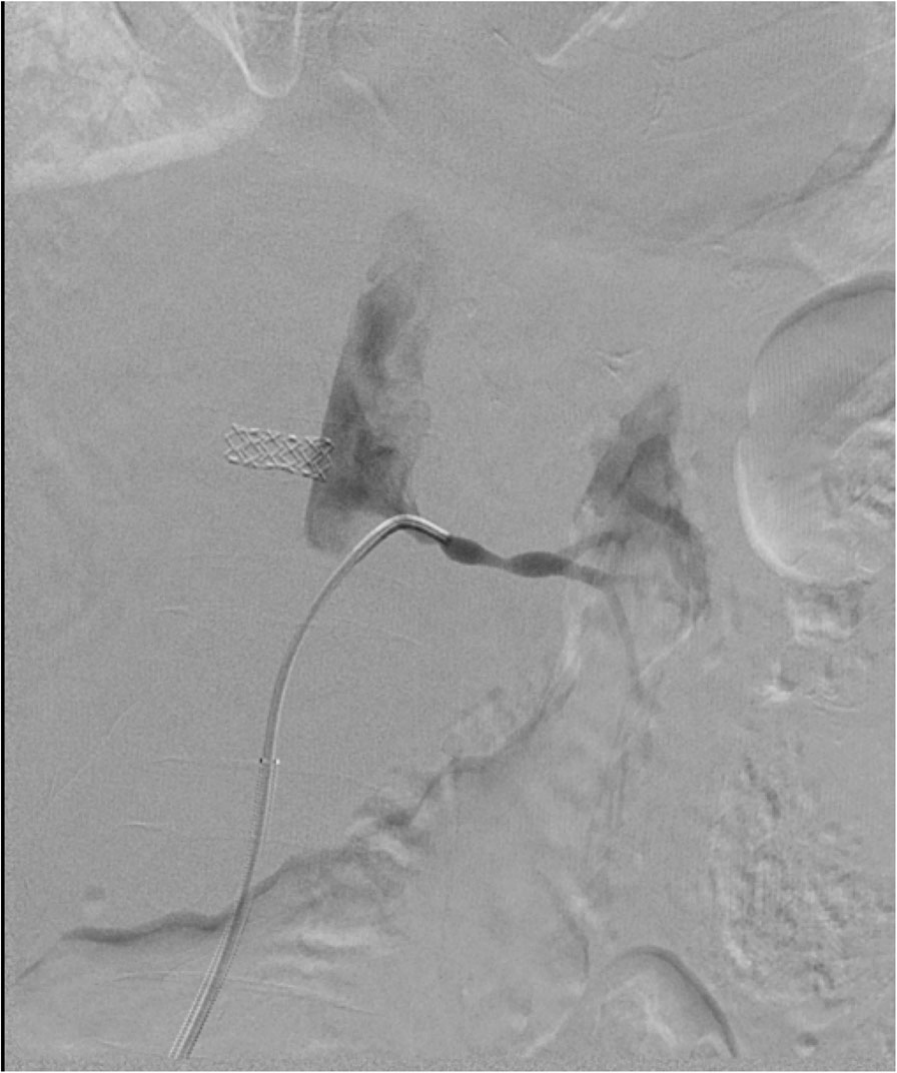
Selective left renal artery angiogram reveals typical cork screw appearance of renal artery in keeping with FMD

**Fig. 5:**
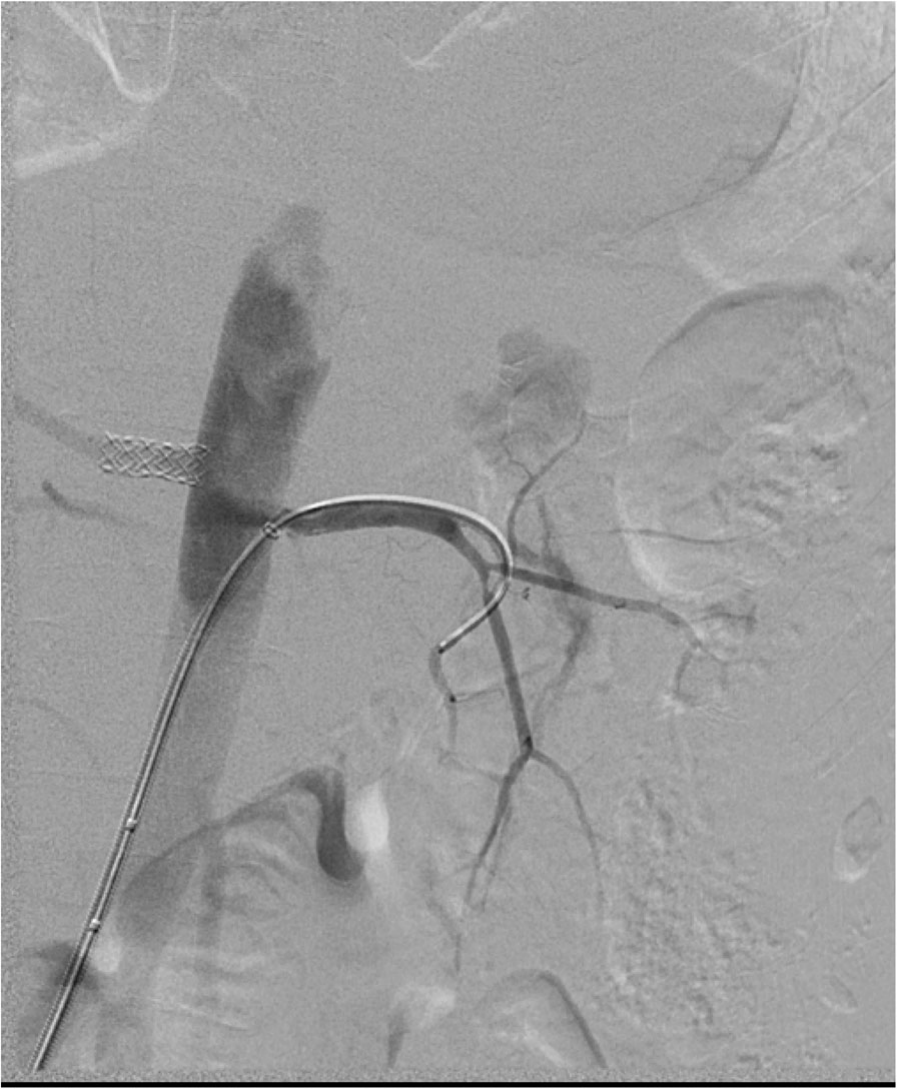
Post angioplasty angiogram showing satisfactory resolution of the FMD

Under local anesthesia, percutaneous femoral access was used to introduce the catheter, and selective tight renal artery catheterization was performed. Selective angiogram of both renal arteries revealed right sided atherosclerotic renal artery stenosis (RAS), treated with insertion of a balloon mounted 6 mm stent and left sided fibromuscular dysplasia (FMD), treated with 5 mm balloon angioplasty (Figs. 1, 2, 3, 4 and 5). The average (of three sessions) intradialytic blood pressure prior to the procedure was 161/81 mmHg. The patient’s average intradialytic blood pressure (six sessions) post intervention was reduced to 135/85 mmHg. There were no further episodes of intradialytic elevation in blood pressure. The reduction in blood pressure has been sustained over 18 months and has improved to the point that labetalol, furosemide and amiloride, have been eliminated from the patient’s antihypertensive regimen. There has been no change in the patient’s goal weight (54–55 kgs) over the last year.

## Discussion and conclusion

Jula Inrig, in an elegantly written review on IDH [1] based on prior studies compiled the existing definitions: an increase in mean arterial blood pressure (MAP) ≥ 15 mmHg during or immediately after hemodialysis [2], an increase in systolic BP (SBP) > 10 mmHg from pre to postdialysis [3, 4], IDH is reported to impact 5–15% of hemodialysis patients [2] and occurs more frequently in older patients with lower serum creatinines, lower dry weights, and on multiple antihypertensive medications [1]. African Americans were more likely to exhibit an increase in SBP pre to postdialysis, despite similar volume of ultrafiltration [5, 6]. IDH is associated with higher likelihood of hospitalization and mortality in ESRD [7]. The mechanisms leading to IDH are poorly understood and the common contributors are thought to be factors that lead to vasoconstriction or stimulation of the sympathetic nervous system, endothelial dysfunction and other factors (inadequate volume removal coupled with higher dialysate sodium use [8], activation of the renin–angiotensin system [1], high calcium dialysate that increases myocardial contractility [9], peripheral resistance [9] and low potassium dialysate [10]). The patient was on six antihypertensives which were chosen based on drug dialyzability and empiric evidence. In a recently published study from July 2018, both nebivolol and irbesartan were found to be valuable in reducing post dialysis and 24-h blood pressure in patients with IDH [11].

Prior investigations of IDH have suggested volume overload may be a key contributor to its pathogenesis. However, the efficacy of aggressive UF during dialysis in patients with IDH is unclear. While there is data to support such a move with normalization of blood pressure [12], there is also data that shows that aggressive UF makes no difference to the outcomes [13]. In our patient we attempted to optimize goal weight by tight fluid control, salt restriction, increased dialysis times/frequency, and decreasing sodium in the dialysate. Modest reductions in goal weight made no impact on IDH and aggressive approach led to cramps that often lasted 6–8 h.

As there was no improvement in blood pressure with conventional measures, we looked at secondary causes of hypertension in dialysis patients [14]. They include: renovascular disease, primary hyperaldosteronism, obstructive sleep apnea (OSA), thyroid disease, pheochromocytoma and renin secreting tumors [14]. The patient underwent a non-contrast CT scan which failed to identify adrenal adenomas, her BMI was 18 and there was no clinical history suggestive of OSA, and no biochemical evidence of thyroid disease based on TSH levels. Failing to improve IDH by conventional approaches and acting upon literature suggesting occult renovascular disease in some patients on hemodialysis [15, 16], we pursued an angiographic approach to further interrogate the possibility of renal artery stenosis. Upon performing a bilateral renal angiogram, we observed evidence of right sided atherosclerotic renal artery stenosis and left sided FMD. Following successful bilateral angioplasty of the blood vessels and an additional 6 mm stent on the right side, she experienced a sustained reduction in pre, intra and post dialysis blood pressure.

The mechanisms of hypertension in FMD appear to overlap with the proposed pathogenic mechanisms for IDH. FMD is a nonatherosclerotic, noninflammatory vascular disease that may result in arterial stenosis, occlusion, aneurysm, or dissection. Reduction in arterial lumen and the subsequent reduction in perfusion pressure by a unifocally or multifocally stenosed artery leads to activation of the renin–angiotensin–aldosterone system, with volume expansion and hypertension. We hypothesize that ultrafiltration worsens the gradient of renal artery stenosis leading to additional upregulation of the renin angiotensin aldosterone system and subsequent further upsurge in blood pressure. This is the first reported case, to our knowledge, of concurrent FMD in an individual with ESRD from ADPKD. Studies of renal angiograms in potential renal donors revealed a prevalence of asymptomatic FMD at close to 4% [17] with an average age of 50 years [17]. No studies have yet been done to study the prevalence of FMD in the dialysis population. We hope that our case report encourages care givers to consider investigating for renovascular disease in patients with refractory IDH.

Bilateral renal angioplasty in our patient led to impressive reductions in blood pressure and allowed a decrease of 2 drugs. Our case illustrates that in patients with ESRD and difficult to treat IDH, there is value in imaging the renal arteries to evaluate for renal artery stenosis.

## List of abbreviations

ADPKD: Autosomal dominant polycystic kidney disease
AV: Arterio-venous
FMD: Fibromuscular dysplasia
HD: Hemodialysis
IDH: Intradialytic hypertension
MAP: Mean arterial blood pressure
OSA: Obstructive sleep apnea
RAS: Renal artery stenosis
RRT: Renal replacement therapy
SBP: Systolic BP
UF: Ultrafiltration
